# A Case of Primary Hypoparathyroidism Presenting with Acute Kidney Injury Secondary to Rhabdomyolysis

**DOI:** 10.1155/2016/3240131

**Published:** 2016-03-10

**Authors:** Abdullah Sumnu, Zeki Aydin, Meltem Gursu, Sami Uzun, Serhat Karadag, Egemen Cebeci, Savas Ozturk, Rumeyza Kazancioglu

**Affiliations:** ^1^Department of Nephrology, Istanbul Medipol University, 34214 Istanbul, Turkey; ^2^Department of Nephrology, Haseki Training and Research Hospital, 34130 Istanbul, Turkey; ^3^Department of Nephrology, Bezmialem Vakif University Medical Faculty, 34093 Istanbul, Turkey

## Abstract

Hypoparathyroidism is the most common cause of symmetric calcification of the basal ganglia. Herein, a case of primary hypoparathyroidism with severe tetany, rhabdomyolysis, and acute kidney injury is presented. A 26-year-old male was admitted to the emergency clinic with leg pain and cramps, nausea, vomiting, and decreased amount of urine. He had been treated for epilepsy for the last 10 years. He was admitted to the emergency department for leg pain, cramping in the hands and legs, and agitation multiple times within the last six months. He was prescribed antidepressant and antipsychotic medications. He had a blood pressure of 150/90 mmHg, diffuse abdominal tenderness, and abdominal muscle rigidity on physical examination. Pathological laboratory findings were as follows: creatinine, 7.5 mg/dL, calcium, 3.7 mg/dL, alanine transaminase, 4349 U/L, aspartate transaminase, 5237 U/L, creatine phosphokinase, 262.000 U/L, and parathyroid hormone, 0 pg/mL. There were bilateral symmetrical calcifications in basal ganglia and the cerebellum on computerized tomography. He was diagnosed as primary hypoparathyroidism and acute kidney injury secondary to severe rhabdomyolysis. Brain calcifications, although rare, should be considered in dealing with patients with neurological symptoms, symmetrical cranial calcifications, and calcium metabolism abnormalities.

## 1. Introduction

Symmetric calcification of the basal ganglia identified radiographically occurs in a variety of familial and nonfamilial conditions. Primary Familial Brain Calcifications (PFBC), which were known by many names previously, including Fahr disease and striopallidodentate calcinosis, are a genetic disease characterized by various mutations in four separate genes and autosomal dominant inheritance [1–4]. PFBC may present with various psychiatric and neurological symptoms [5]. On the other hand, many secondary causes, either infectious, toxic, or metabolic, have been described to cause symmetrical basal ganglion calcifications and so are in the differential diagnosis of PFBC [6]. Parathyroid diseases such as hypoparathyroidism, pseudohypoparathyroidism, and pseudo-pseudohypoparathyroidism are in the forefront among the metabolic causes. Idiopathic or postsurgical hypoparathyroidism is the most common cause of symmetric calcification of the basal ganglia [7–9]. Herein, a case of primary hypoparathyroidism with severe tetany, rhabdomyolysis, and acute kidney injury (AKI) is presented.

## 2. Case

A twenty-six-year-old male was admitted to the emergency clinic with pain and cramps in the legs, nausea, vomiting, and a decrease in the amount of urine. He had a head trauma due to a fall from a height 15 years ago requiring treatment for 45 days in the intensive care unit and had been treated for epilepsy for the last 10 years. He had no epileptic attack for the last eight months. He was admitted to the emergency department for leg pain, cramping in the hands and legs, and agitation at recurrent times within the last six months. He was prescribed antidepressant and antipsychotic medications. He had trauma to various sites of the body during these episodes of agitation and convulsions. He was using phenytoin, levetiracetam, sertraline, and olanzapine. He had no history of smoking or alcohol use. Past medical and family histories were unremarkable.

**Figure 1:**
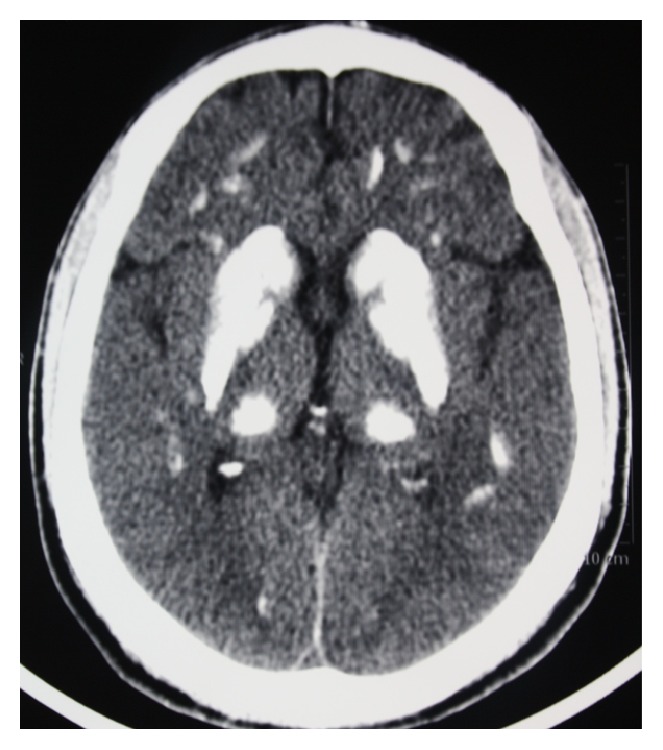
Bilateral symmetrical calcifications on cranial CT.

He had a blood pressure of 150/90 mmHg, diffuse abdominal tenderness, and abdominal muscle rigidity on physical examination. Major laboratory findings are presented in Table 1. Pathological laboratory findings were as follows: creatinine, 7.5 mg/dL (0.7–1.2), calcium, 3.7 mg/dL (8.8–10.2), alanine transaminase (ALT), 4349 U/L (<41), aspartate transaminase (AST), 5237 U/L (<40), creatine phosphokinase (CPK), 262,000 U/L (39–308), and parathyroid hormone, 0 pg/mL (15–65). There were bilateral symmetrical calcifications in basal ganglia and the cerebellum on cranial CT (Figure 1).

He was diagnosed as primary hypoparathyroidism and AKI secondary to rhabdomyolysis. His antipsychotic medication (olanzapine) was stopped and the dosage of antiepileptic drugs was decreased. Because of continuing oliguria despite proper hydration, he required five sessions of hemodialysis. Calcium was replaced parenterally due to nausea and vomiting, followed by oral calcium and calcitriol treatment after resolution of these symptoms with the doses titrated according to calcium levels and hypocalcemic symptoms. Within one week, symptoms of hypocalcemia diminished and rhabdomyolysis recovered with CPK levels returning to normal followed by polyuria and full recovery of renal functions. Nifedipine, metoprolol, and spironolactone were administered for the treatment of hypertension. Creatinine, CPK, AST, and ALT levels were within normal levels 15 days after admission to the hospital. The need for antihypertensive agents was abolished with normalization of renal functions. He was discharged with a treatment protocol of oral calcium (9 gr/day), calcitriol (2 *μ*g/day), and the antiepileptic agents and was advised to be followed up in the endocrinology outpatient clinic. He was brought to the emergency clinic 15 days after discharge with confusion. His serum calcium level was 16 mg/dL. He was hospitalized again with the diagnosis of iatrogenic hypercalcemia and was treated with intravenous furosemide and proper hydration. After gaining normal calcium levels, he was discharged with reorganized oral calcium and calcitriol treatment. He is asymptomatic with normal calcium levels for the last two months.

## 3. Discussion

Brain calcifications were first described in 1935 as radiographic images of calcinosis on direct cranium X-rays [10]. They have been associated with more than fifty clinical syndromes [11]. Some of the secondary reasons for brain calcifications are presented in the following list. The term “Fahr disease” is used in the literature to define idiopathic or familial brain calcifications, while the term “Fahr syndrome” is used mostly for secondary brain calcifications. However, it is recommended not to use these terms because these are misnomers. Recently, the term “PFBC” has been used for familial cases and “primary (formerly idiopathic) and secondary brain calcifications” for other brain calcifications [1]. The reason for brain calcifications was idiopathic hypoparathyroidism in the present patient.

*Some of the Secondary Reasons of Brain Calcifications.* Some of the secondary reasons of brain calcifications are listed as follows:

- Parathyroid diseases:
  - Hypoparathyroidism.
  - Hyperparathyroidism.
  - Pseudohypoparathyroidism.
  - Pseudo-pseudohypoparathyroidism.
- Hypothyroidism.
- Perinatal asphyxia.
- Intoxications:
  - Carbon monoxide.
  - Lead.
- Infectious diseases:
  - Epstein-Barr virus.
  - Cytomegalovirus.
  - Human immunodeficiency virus.
  - Toxoplasmosis.
- Systemic lupus erythematosus.
- Acute lymphocytic leukemia.
- Down syndrome.
- Radiotherapy and methotrexate treatment.
- Sturge-Weber syndrome, tuberous sclerosis, and neurofibromatosis.

Olanzapine related neuroleptic malignant syndrome [12], severe tetany, and trauma during agitation episodes have been considered in the differential diagnosis of rhabdomyolysis in our case. Lack of fever was not consistent with a diagnosis of malignant neuroleptic syndrome, but it is reported that the disease can have an atypical course and fever may appear later [13].

Epilepsy is not a typical neurological presentation of PFBC. But there are a considerable number of case reports with coexistence of epilepsy and brain calcifications secondary to hypoparathyroidism [8, 14, 15]. The roles of hypocalcemia and intracerebral calcifications in the occurrence of epilepsy in hypoparathyroidism are not entirely clear. El Otmani et al. [16] analyzed retrospectively the neurological and clinical findings of 12 cases with secondary brain calcifications (9 of them were hypoparathyroidism and 3 of them were pseudohypoparathyroidism) during a follow-up period of 13 years. In this report, all patients had a tetany, 60% had epilepsy, 50% had behavioral changes, and response to calcium therapy was excellent for all these events [16]. Although the present patient had a history of head trauma and continuing convulsive attacks until the last eight months despite antiepileptic treatment, the presence of hypocalcemic symptoms like tetany and agitation may support the idea that the epileptic attacks were related to primary hypoparathyroidism and brain calcifications. Moreover, he had no attacks after the treatment of hypocalcemia even though the doses of antiepileptic agents were reduced. The prescribed antipsychotic drugs may be a clue to the possible past psychiatric symptoms of brain calcifications.

The present case is different from the other reported brain calcification cases in that the patient presented with deep hypocalcemia, severe rhabdomyolysis, and AKI. Calcium metabolism disorders should be investigated in patients with symmetrical cranial calcifications and recurrent attacks of tetany and convulsions.

## Figures and Tables

**Table 1:** Laboratory data of the patient at admission and at discharge.

Parameter (unit)	Result at admission	Result at discharge	Normal range
Leucocyte (/mm^3^)	14300	8800	4400–11300
Hemoglobin (g/dL)	12.6	11.9	14–17.5
Hematocrit (%)	37	34	41–51
Platelet (/mm^3^)	155000	199000	152–362
Urine sediment	8–10 erythrocytes/field	—	<2
Proteinuria (mg/day)	435	210	<150
Urea (mg/dL)	181	38	17–48
Creatinine (mg/dL)	7.5	1.1	0.7–1.2
LDH (U/L)	19195	241	135–225
Sodium (mmol/L)	130	138	135–145
Potassium (mmol/L)	5.3	4.7	3.5–5.1
Calcium (mg/dL)	3.7	8.8	8.8–10.2
Phosphorus (mg/dL)	6.2	3.1	2.6–4.5
PTH (pg/mL)	0	2	15–65
CPK (U/L)	262000	299	39–308
ALT (U/L)	4349	24	<41
AST (U/L)	5237	35	<40
Albumin (g/dL)	2.8	3.6	3.5–5.2

LDH: lactate dehydrogenase; PTH: parathyroid hormone; CPK: creatine phosphokinase; ALT: alanine transaminase; AST: aspartate transaminase.
